# Supplementary material for: Spectrum of sexual partner types among adults screened for sexually transmitted infections in the Eastern Cape, South Africa
Source: PLoS One. 2025 May 7;20(5):e0323414. doi: 10.1371/journal.pone.0323414 (PMC12057859; doi:10.1371/journal.pone.0323414)
Supplement: S1 Table — (PDF) [file pone.0323414.s001.pdf]

Supplemental Table 1 (S1 Table). LUSTRUM's 4 partner types and Xpert CT/NG positivity

	Male (n=500)		Female (n=400)		
**Partner definition**	**CT Positive**	**CT Negative**	**CT Positive**	**CT Negative**	***p***
Established partner	34 (10.9)	279 (89.1)	27 (9.4)	259 (90.6)	-
New partner	6 (14.0)	37 (86.0)	3 (12.5)	21 (87.5)	0.53
Occasional partner	23 (12.0)	169 (88.0)	8 (12.7)	55 (87.3)	0.85
Once-off partner	10 (10.9)	82 (89.1)	3 (17.7)	14 (82.4)	0.65
	**NG Positive**	**NG Negative**	**NG Positive**	**NG Negative**	***p***
Established partner	17 (5.4)	296 (94.6)	24 (8.4)	262 (91.6)	-
New partner	8 (18.6)	35 (81.4)	3 (12.5)	21 (87.5)	0.01
Occasional partner	17 (8.6)	175 (91.2)	5 (7.9)	58 (92.1)	0.93
Once-off partner	4 (4.4)	88 (95.7)	2 (11.8)	15 (88.2)	0.13
