# Supplementary material for: Spectrum of sexual partner types among adults screened for sexually transmitted infections in the Eastern Cape, South Africa
Source: PLoS One. 2025 May 7;20(5):e0323414. doi: 10.1371/journal.pone.0323414 (PMC12057859; doi:10.1371/journal.pone.0323414)
Supplement: Attachment — Submitted filename: Response to Reviewers_19 July 2024.pdf [file pone.0323414.s002.pdf]

Reviewer Comment	Author's Revision
**Editorial Comments**	
"Thank you for submitting your work to PLOS ONE. Before we can proceed,<br><br>1) We have noticed that you have provided two dates for recruitment (see lines Abstract of online submission form and 79). Please clarify and ensure that you report in the Methods section the day, month and year of the start and end of the recruitment period for this study (recruitment refers to when the participants were formally enrolled into the study) in DD/MM/YYYY format.	We thank the editorial team for their queries.<br><br>We have provided clarity and consistency on the recruitment dates for participants in the Abstract in the online submission (February – August 2023), and reported the days, months and year for enrolment into the study as DD/MM/YYYY in the Methods (Line 79).
2) During our evaluation of the documents provided, we noted that your ethics approval letter did not cover the entire date range for participant recruitment. Before we can proceed further with the submission, please provide the original ethics document / ethics approval extension document(s) for the study. If the document is in another language, please also provide an English translation.	In addition to the protocol amendment this speaks to this study, and the prior annual renewal from 2023-2024, we have uploaded the original ethics approval certificate and the approval extension for 2022-2023, that covers the entire date range for participant recruitment of this study.
