# Supplementary material for: Spectrum of sexual partner types among adults screened for sexually transmitted infections in the Eastern Cape, South Africa
Source: PLoS One. 2025 May 7;20(5):e0323414. doi: 10.1371/journal.pone.0323414 (PMC12057859; doi:10.1371/journal.pone.0323414)
Supplement: Attachment — Submitted filename: Response to Reviewers_26March2025.docx [file pone.0323414.s003.docx]

| **JOURNAL REQUIREMENTS** | |
| --- | --- |
| 1. 1. Please ensure that you include a title page within your main document. You should list all authors and all affiliations as per our author instructions and clearly indicate the corresponding author. | Thank you for this information. We apologize that the title page was not included in the main document. The original title page has now been merged in the main manuscript file. |
| 2. It appears that your ORCiD iD has not been validated in your Editorial Manager account and we are unable to proceed until that step is complete.   To validate your ORCiD iD in Editorial Manager, please follow the steps below:   a. In your Editorial Manager account, please go to ‘Update my Information’ (in the upper left-hand corner of the main menu), and click on the Fetch/Validate link next to the ORCiD field. b. This link will take you to the ORCiD site and allow you to create a new iD or authenticate a pre-existing iD in Editorial Manager. | The ORCiD ID for the corresponding author has now been linked to the Editorial Manager as per the requested steps. |
